# Eliminating the Interference of Oxygen for Sensing Hydrogen Peroxide with the Polyaniline Modified Electrode

**DOI:** 10.3390/s8128237

**Published:** 2008-12-12

**Authors:** Yesong Gu, Chien-Chung Chen

**Affiliations:** Department of Chemical and Materials Engineering, Tunghai University, Taichung 40704, Taiwan

**Keywords:** Amperometric electrode, polyaniline, hydrogen peroxide, oxygen scavenger

## Abstract

Polyaniline (PANI) has been shown to possess excellent catalytic activity toward oxygen reduction, however, this molecule may interfere with the electrochemical measurement of other targets when using a polyaniline modified platinum (PANI/Pt) electrode. In this study, we have demonstrated the considerable effects of dissolved oxygen on the sensing of hydrogen peroxide with the PANI/Pt electrode. Accordingly, we proposed a strategy to eliminate the influence of dissolved oxygen with oxygen scavengers. Our results indicated that as an oxygen scavenger sodium thiosulfate was very effective in the removal of dissolved oxygen from the sample solution, and had negligible effect on the quantification of hydrogen peroxide when its applied concentration was below 1 mM.

## 1. Introduction

Polyaniline (PANI) has received considerable attention in biosensor fabrication because of its comparative conductivity, unique optical quality, and thermal stability [1-6]. The synthetic porous PANI matrix provides a suitable microenvironment for the immobilization of biocomponents, including enzymes, DNA, antibodies, and cells [7-14]. PANI-based biosensors have been successfully applied in many fields, in particular, monitoring in biological fluids of compounds with clinical significance, such as urea [15, 16], uric acid [17, 18], hydrogen peroxide (H_2_O_2_) [19, 20], glucose [21, 22], cholesterol [17, 23] and choline [24]. Most biosensors rely upon the target being involved in oxidation-reduction reactions catalyzed by oxidases, such as uricase, glucose oxidase, cholesterol oxidase, and cholin oxidase, where H_2_O_2_ is a common co-product. Consequently, the quantification of H_2_O_2_ becomes an alternative strategy to measure these target molecules.

On the other hand, the reduction of oxygen by the conductive PANI film has also been well recognized and investigated intensively [25-29]. Although PANI as a catalyst for oxygen reduction has potential applications in battery and fuel-cell development [27, 30-32], oxygen reduction may bring about unexpected interference while electrochemically measuring the above mentioned biomolecules with a PANI modified electrode. For this reason, a routine measurement of H_2_O_2_ is normally carried out under an anaerobic environment, and the sample is diluted with a large volume of buffer solution that has been deoxygenated with pure N_2_[33]. However, it may be inconvenient for those containing lesser amounts of H_2_O_2_. The direct deoxygenation of sample solutions is also not a suitable solution due to the instability and volatility of H_2_O_2_. Consequently, the remaining oxygen may lead to overestimation of the final H_2_O_2_ concentration.

In order to establish a procedure that is able to eliminate the interference of oxygen on sensing H_2_O_2_, in this study, we investigated the electrochemical response of oxygen with the PANI modified Pt electrode. Our results demonstrated that both air-saturated and oxygen-saturated solution exhibited significant interference on sensing H_2_O_2_, but the dissolved oxygen could be effectively removed by the addition of oxygen scavengers, such as sodium thiosulfate and ascorbic acid. In addition, oxygen scavengers with various concentrations were examined and the recommended concentration was below 1 mM because of the negligible effects on the reduction of H_2_O_2_.

## 2. Experimental

### 2.1. Chemicals

Hydrogen peroxide (35%, v/v) and aniline monomer were obtained from Merck (KGaA Darmstadt, Germany). Sodium thiosulfate and ascorbic acid were purchased from Sigma-Aldrich (Saint Louis, MO, USA). All other reagents used for buffer and standard solution preparation were purchased from various commercial sources and were of analytical grade.

### 2.2. Electrochemical apparatus

A PC-controlled CHI621B electrochemical analyzer (CH Instruments, Austin, USA) was employed to run cyclic voltammetric experiments for electrode preparation and electrochemical measurement. All experiments were proformed in a miniature electrochemical cell using a modified Pt electrode (area: 0.28 cm^2^) as the working electrode, a platinum wire as the auxiliary electrode, and a Ag/AgCl 3M NaCl electrode as the reference electrode.

### 2.3. Preparation of electrodes

The PANI/Pt electrode was constructed as previously described [22]. On a ceramic plate (area: 2.00 cm^2^), platinum was sputtered with a shadow mask desired (area: 0.28 cm^2^) for 10 min on a sputter instrument (JFC-1200, JEOL, Japan). The Pt/ceramic electrode was washed with 3 M NaOH and 3 M HCl, rinsed with water, and finally dried under 50°C for one hour. A certain amount of aniline was then electropolymerized onto the Pt/ceramic base by immersing the working electrode in a solution containing 1 M HCl and 0.1 M aniline, whilst the potential was swept from 0.0 to 1.0 V for four cycles under ambient conditions. The PANI/Pt electrodes thus fashioned were then immersed in a phosphate-buffered saline solution PBS (pH 4.0) and reduced at -0.5 V for 20 min to remove any remaining chloride ions that were possibly embedded in the polymer matrix. It was then oxidized in the same PBS buffer at 0.6 V for 10 min. The surface morphologies of the electrodes were visualized by an ABT-150S Scanning Electron Microscopic (SEM, TOPCON Corp., Tokyo, Japan).

### 2.4. Electrochemical measurements

The oxidation and reduction of H_2_O_2_ on a Pt or PANI/Pt electrode were quantified with cyclic voltammetry in 0.1 M PBS buffer (pH 6.2). The buffer had undergone deoxygenation with highly pure nitrogen for 20 min before a certain amount of H_2_O_2_ was added. During the calibration, pure nitrogen gas was gentle purged on the surface of the sample solution to create an anaerobic atmosphere. To investigate the reduction of oxygen on a Pt or PANI/Pt electrodes, the solution was purged with pure oxygen for 30 minutes to reach the saturate concentration. The fresh prepared oxygen scavenger, sodium thiosulfate or ascorbic acid, was added into the solution before the electrochemical measurement.

## 3. Results and discussion

### 3.1. Oxidation and reduction on a Pt electrode

**Figure 1.**
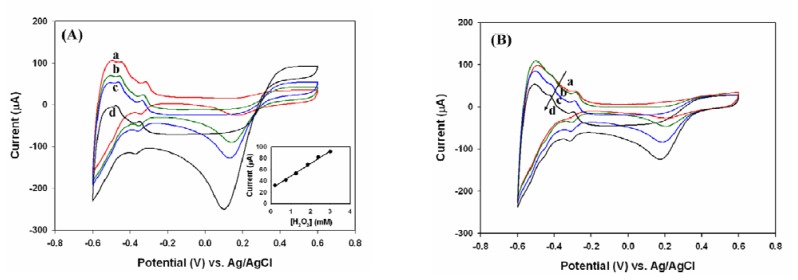
Cyclic voltammograms of a Pt electrode in the potential window of -0.6 ∼ 0.6 V with the scan rate of 0.2 V/s in 0.1 M phosphate buffer (pH 6.2). (A) The solutions were degassed before H_2_O_2_ was added. The concentration of H_2_O_2_ was 0, 0.748, 1.25, and 2.99 mM for lines (a) to (d), respectively, and the inset indicated the linear correlation of anodic peak current at 0.6 V with the concentration of H_2_O_2_. (B) The solutions were degassed before the oxygen-saturated sample was added, and the concentration of O_2_ was 0, 0.43, 0.86, and 1.3 mM for lines (a) to (d), respectively.

It is well known that platinum is able to catalyze the electrochemical oxidation of H_2_O_2_[34]. Within our selected potential range between -0.6 to 0.6 V, the addition of H_2_O_2_ gave rise to a near 0.6 V anodic response on the bare Pt electrode (Figure 1A), which resulted from the oxidation of H_2_O_2_*vs.* Ag/AgCl in 0.1 M phosphate buffer solution (pH 6.2), as shown in reaction (1):

(1) $${\text{H}}_{2}{\text{O}}_{2}\overset{Pt}{⟶}{\text{O}}_{2}+2{\text{H}}^{+}+2e^{-}$$

Meanwhile, a major cathodic peak between 0.1 ∼ 0.2 V was observed, whereas the potential shift slightly towards negative with the increase of H_2_O_2_ concentration. Applied the same electrode to an oxygen-saturated solution with the same potential window, the cathodic peak near 0.2 V was also visible with similar peak shift, but not the reduction of H_2_O_2_ near 0.6 V (Figure 1B). Therefore, the cathodic response to H_2_O_2_ in Figure 1A was possibly associated with the further reduction of oxygen on the Pt electrode, in which one-step four electron pathway was proposed as reaction (2) [35]:

(2) $${\text{O}}_{2}+4{\text{H}}^{+}+4e^{-}\overset{Pt}{⟶}2{\text{H}}_{2}\text{O}$$

The potential shift in Figure 1A also indicated an increase in the local concentration of oxygen on the Pt electrode. Both anodic and cathodic responses at 0.6 V and between 0.1∼0.2 V in Figure 1A were strongly dependent on the H_2_O_2_ concentration. The inset of Figure 1A shows the linear correlation between the anodic peak current at 0.6 V and the concentration of H_2_O_2_ over the 0∼2.5 mM range (sensitivity: 79.11 μA mM^-1^·cm^-2^, R^2^ = 0.996). However, under our experimental conditions bubbles were observed on the surface of electrode when the H_2_O_2_ concentration was higher than 2.5 mM indicating the local concentration of oxygen had exceeded its saturation point.

**Figure 2.**
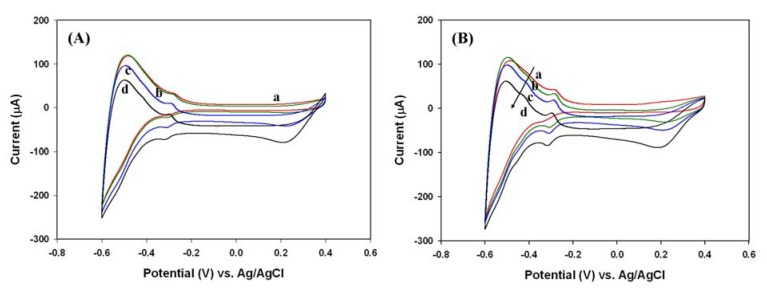
Cyclic voltammograms of a Pt electrode in the potential window of -0.6 ∼ 0.4 V with the scan rate of 0.2 V/s in 0.1 M phosphate buffer (pH 6.2). (A) The solutions were degassed before H_2_O_2_ was added. The concentration of H_2_O_2_ was 0, 0.748, 1.25, and 2.99 mM for lines (a) to (d), respectively. (B) The solutions were degassed before the oxygen-saturated sample was added, and the concentration of O_2_ was 0, 0.43, 0.86, and 1.3 mM for lines (a) to (d), respectively.

By narrowing the potential window to -0.6 ∼ 0.4 V, the above redox responses to H_2_O_2_ near 0.2 and 0.6 V were reduced significantly (Figure 2A). The small cathodic peak near 0.2 V with high concentration of H_2_O_2_ was likely associated with the reduction of oxygen that was formed from the partial decomposition of H_2_O_2_ on the Pt electrode. Similar cathodic peak was also observed while sensing the oxygen-saturated solution with the potential window of -0.6∼0.4 V (Figure 2B). Accordingly, the potential window of -0.6∼0.4 V was employed for further investigation.

### 3.2. Oxidation and reduction of H_2_O_2_ on a PANI/Pt electrode

**Figure 3.**
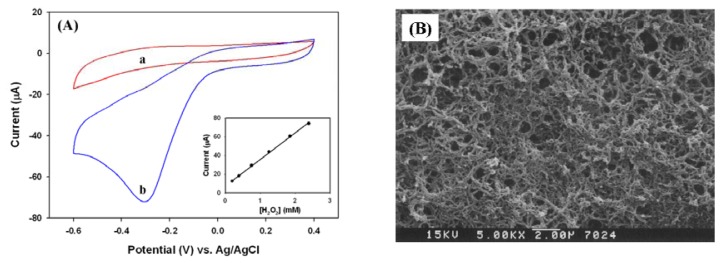
(A) Cyclic voltammograms of PANI/Pt electrode (a) in the absence of H_2_O_2_ (red) and (b) in the presence of 2.39 mM of H_2_O_2_ (blue). Inset indicated the linear correlation of cathodic peak current at -0.32 V with the concentration of H_2_O_2_. (B) The SEM image of PANI film on the Pt electrode.

A PANI film was then electrochemically synthesized on the surface of Pt electrode to form a PANI/Pt electrode. As shown in Figure 3A (red line a), the modified electrode completely suppressed the hydrogen adsorption-desorption redoxs between -0.5 and -0.6 V that was seen in Figures 1 and 2, thereby the PANI film effectively minimizing the background influences from the Pt electrode. Alternatively, H_2_O_2_ was electrochemically reduced with a peak potential centered at -0.32 V (blue line b in Figure 3A) by the following reaction (3):

(3) $${\text{H}}_{2}{\text{O}}_{2}+2{\text{H}}^{+}+2e^{-}\overset{\text{PANI}}{⟶}2{\text{H}}_{2}\text{O}$$

The inset of Figure 3A shows the linear correlation between the cathodic peak current near -0.32 V and the concentration of H_2_O_2_ in the range of 0 ∼ 2.5 mM (sensitivity: 101.14 μA mM^-1^·cm^-2^, R^2^ = 0.999). The SEM image indicated the typical sponge-like PANI film (Figure 3B). Due to the significant surface area provided by the microstructure of PANI film, the major cathodic response was extremely sensitive to the concentration of H_2_O_2_. Therefore, the PANI modified Pt electrode provided better sensitivity and selectivity than a bare Pt electrode, and had potential applications for fabricating H_2_O_2_ involved biosensors.

### 3.3. The effect of oxygen on sensing H_2_O_2_

**Figure 4.**
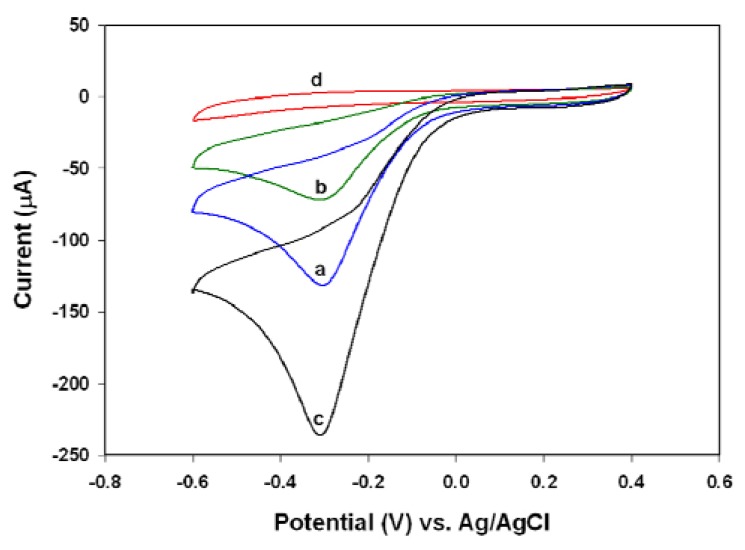
Cyclic voltammograms of PANI/Pt electrode in 0.1 M phosphate buffer (pH 6.2) containing 2.39 mM H_2_O_2_ , where (a) had no further pretreatment (blue), (b) was purged with N_2_ for 10 minutes (green), and (c) was purged with pure O_2_ for 10 minutes (black). There was no H_2_O_2_ added for (d) (red).

Considering the catalytic activity of PANI towards oxygen reduction [29], the electrochemical measurement of H_2_O_2_ with PANI/Pt electrode was normally performed under an anaerobic atmosphere with solution that had undergone a deoxygenation procedure. As depicted in Figure 4, a significant cathodic peak at -0.32 V was observed for 2.39 mM H_2_O_2_ in 0.1 M PBS solution (pH 6.2) without any deoxygenation procedure (blue curve a in Figure 4). Purging the solution with pure nitrogen gas for ten minutes before the addition of H_2_O_2_ decreased the maximum cathodic response by about 1.82-fold (green curve b in Figure 4). In contrast, purging with pure oxygen increased the maximum cathodic response by about 1.80-fold (black curve c in Figure 4). Therefore, the dissolved oxygen may lead to an overestimation of the concentration of H_2_O_2_ measured by PANI/Pt electrode. In particular, the observed peak potential for O_2_ reduction was almost identical to that of H_2_O_2_ reduction under our experimental condition. Accordingly, the reduction of oxygen might go through a two-step four electron pathway on the PANI film, where oxygen was reduced to H_2_O_2_ within the applied potential window followed by immediate reduction of H_2_O_2_ through reactions (4) and (3), respectively:

(4) $${\text{O}}_{2}+2{\text{H}}^{+}+2e^{-}\overset{\text{PANI}}{⟶}{\text{H}}_{2}{\text{O}}_{2}$$

### 3.4. The effect of oxygen scavengers on sensing H_2_O_2_

**Figure 5.**
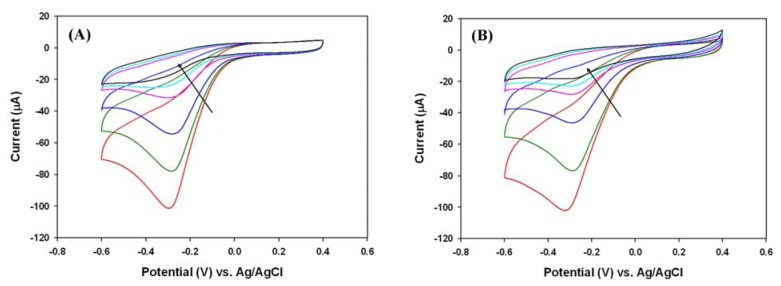
The effects of oxygen scavengers (A) sodium thiosulfate and (B) ascorbic acid. Before addition of oxygen scavenger, the 0.1 M phosphate buffer (pH 6.2) had been purged with pure oxygen for 30 minutes. The arrows indicated the concentrations of scavengers increased in an order of 0, 1, 5, 10, 15, 20 mM.

Although purging with nitrogen is a common option to remove dissolved oxygen, it is not always convenient in practice due to the instability of H_2_O_2_, especially for those samples with relatively low H_2_O_2_ concentrations. To overcome this problem, we modified the common deoxygenation by adding oxygen scavengers, such as sodium thiosulfate (Na_2_S_2_O_3_) and ascorbic acid (C_6_H_8_O_6_). For samples that were oxygen-saturated by purging with oxygen for 30 minutes, both sodium thiosulfate and ascorbic acid were able to effectively decrease the cathodic peak that was corresponding to the dissolved oxygen on the PANI/Pt electrode as shown in Figures 5A and 5B.

**Figure 6.**
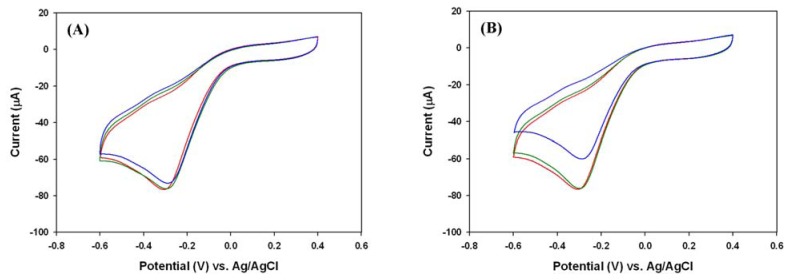
The effects of oxygen scavengers (A) sodium thiosulfate and (B) ascorbic acid on the detection of 2.39 mM H_2_O_2_ in 0.1 M phosphate buffer (pH 6.2). In both Figures, the concentrations of oxygen scavenger were 0 mM (red), 1 mM (green), and 5 mM (blue), respectively.

Sodium thiosulfate and ascorbic acid can also function as mild reducing agents, which may quench H_2_O_2_ in an aqueous solution. For that reason, we investigated whether the reduction of H_2_O_2_ by an oxygen scavenger could directly compete against that catalyzed by PANI in a deoxygenated H_2_O_2_ solution. As shown in Figures 6A and 6B, when the concentration was below 1 mM, both sodium thiosulfate and ascorbic acid showed insignificant effects on the sensing of H_2_O_2_ with the PANI/Pt electrode. Furthermore, the addition of 5 mM ascorbic acid leaded more than 20% reduction of response to 2.39 mM H_2_O_2_, whereas 5 mM sodium thiosulfate caused the lose of less than 5% response. Therefore, the influence of ascorbic acid on the H_2_O_2_ measurement showed more concentration dependence than that of sodium thiosulfate. Nevertheless, the concentration of air-saturated oxygen at the ambient temperature and pressure is approximately 0.26 mM in a buffered solution [36-38], both oxygen scavengers with a concentration below 1mM are capable to completely deprive the air-saturated oxygen according to the stoichiometry of the reaction. Meanwhile, sodium thiosulfate will be more efficient because it has less concentration effect on sensing.

## 4. Conclusions

Polyaniline, a functional electro-conductive polymer, has been successfully applied in biosensor fabrication to eliminate the background interference, to enhance the electrochemical sensitivity, as well as to immobilize biomolecules and cells [14-24]. However, the interference from dissolved oxygen on sensing of H_2_O_2_ with a PANI modified platinum electrode was inevitable, because the electro-state of PANI might be altered by the oxygen reduction on the PANI film [29]. Although purging with nitrogen has been commonly utilized to eliminate the influence of oxygen when sensing H_2_O_2_[33], this is not always feasible in practice. In this study, we further demonstrated that the reduction of oxygen was well catalyzed by PANI film, which might overestimate the electrochemical response to H_2_O_2_. Two oxygen scavengers, such as sodium thiosulfate and ascorbic acid, were examined for their ability to effectively remove the dissolved oxygen. Although H_2_O_2_ has been reported to be directly reduced by both oxygen scavengers, our results demonstrated that the reductions were not comparable to the electrochemical reduction catalyzed by PANI film. As a result, both oxygen scavengers had no significant effect on sensing of H_2_O_2_ by the PANI/Pt electrode as long as their concentrations were below 1 mM. Moreover, sodium thiosulfate had a less concentration dependent than ascorbic acid while sensing H_2_O_2_, hence, was more suitable for practical applications.
